# Supplementary material for: Systematic Methods for Isolating High Purity Nuclei from Ten Important Plants for Omics Interrogation
Source: Cells. 2022 Dec 3;11(23):3919. doi: 10.3390/cells11233919 (PMC9740283; doi:10.3390/cells11233919)
Supplement: Supplementary file 1 [file cells-11-03919-s001.zip › cells-2030101-supplementary.pdf]

Supplementary Table S1. The list of Actin gene primers from maize, rice, tomato, soybean, banana, grape, litchi, citrus, and apple

	**ID**	**Primer**
Maize	J01238.1	TCCACCATCTCTGACAGCCTTGA<br>CAGCCTTCACCATTCCAGTTCCAT
Rice	AK058421	GCCTTGACCATACCAGTTCCATT<br>CCACCACCTCCACCTCCT
Tomato	AB199316.1	GGAATGGGACAGAAGGATGCGTAT<br>GGTGCCTCAGTCAGGAGAACAG
Arabidopsis	NM_112764	CCGCTCTGCTGTTGTGGTGAA<br>ATTGTGCTGGATTCTGGTGATGGT
Soybean	Glyma 18g52780	ATCTTGACTGAGCGTGGTTATTC<br>GCTGGTCCTGGCTGTCTC
Banana	AF285176.1	AGCGAAGCAAGGATGGAGCC<br>GCAACATCGTTCTCAGTGGTGGTA
Grape	AF369524.1	ACCACTACTGCTGAACGGGAAAT<br>GGACTTCTGGACAACGGAATCTCT
Litchi	HQ615689.1	ACCGTATGAGCAAGGAAATCACTG<br>TCGTCGTACTCACCCTTTGAAATC
Citrus	XM_006464503.2	CCAAGCAGCATGAAGATCAA<br>ATCTGCTGGAAGGTGCTGAG
Apple	XM_008393049.3	GGATTTGCTGGTGATGATGCT<br>AGTTGCTCACTATGCCGTGCT

Supplementary Figure S1. Sorted nuclei observed under BD Rhapsody™ Scanner on brightfield.

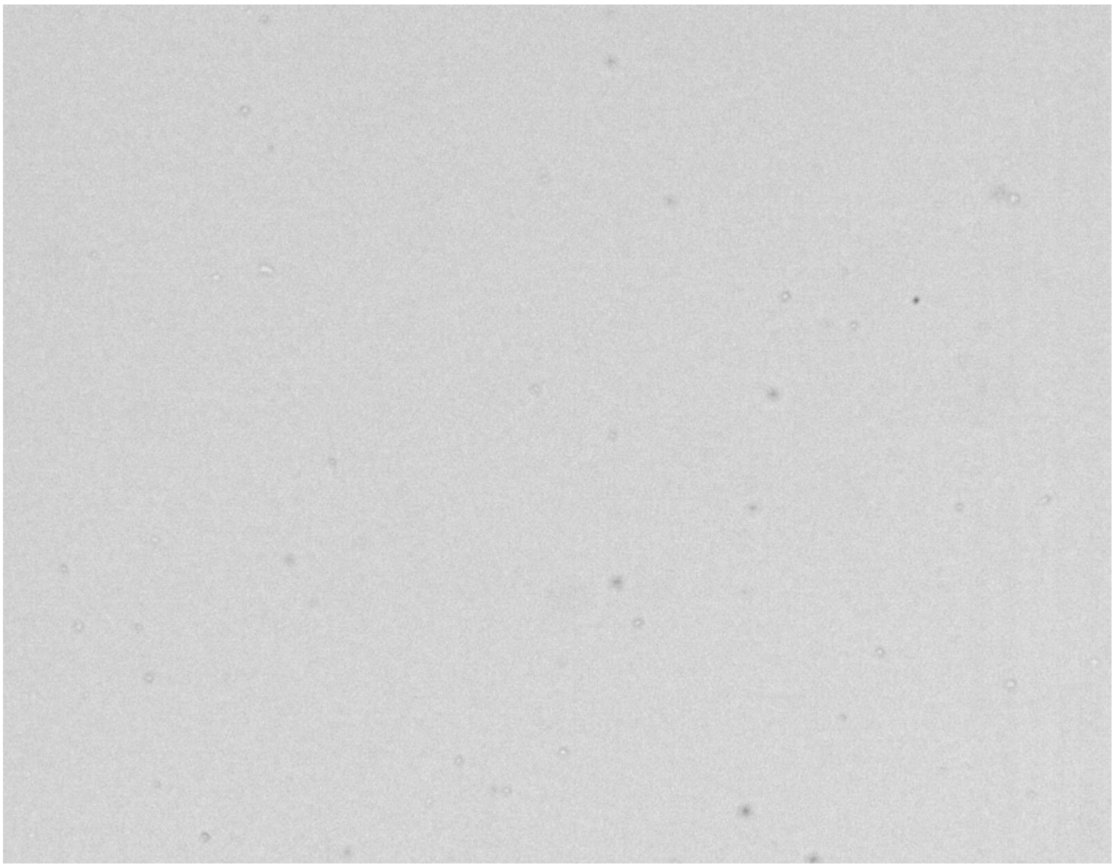

Supplementary Figure S2. Library peak diagram and gel image

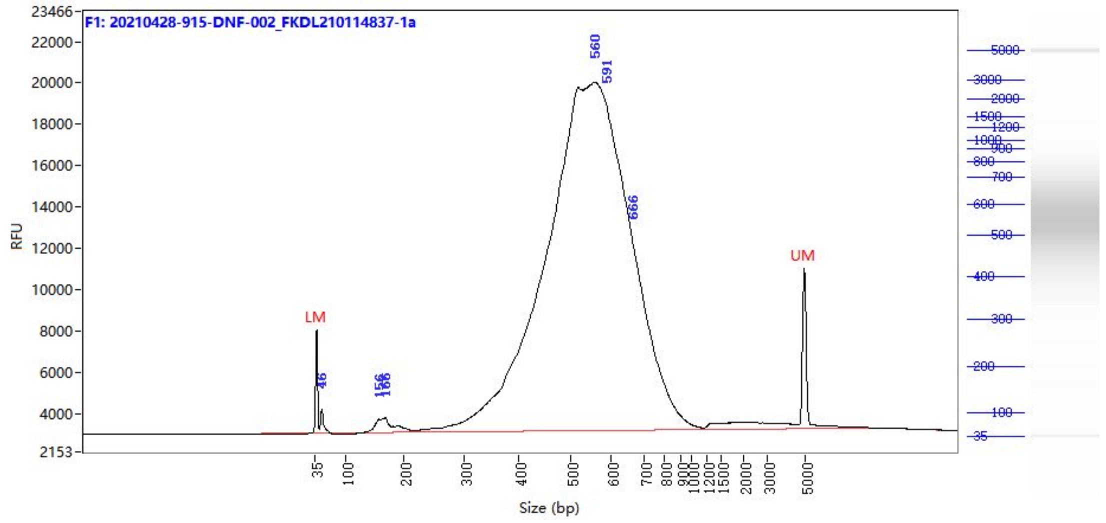

Supplementary Figure S3. t-SNE plot of the litchi bud showing 16 cell clusters Each dot represents a single cell, and the colors portray corresponding cell clusters.

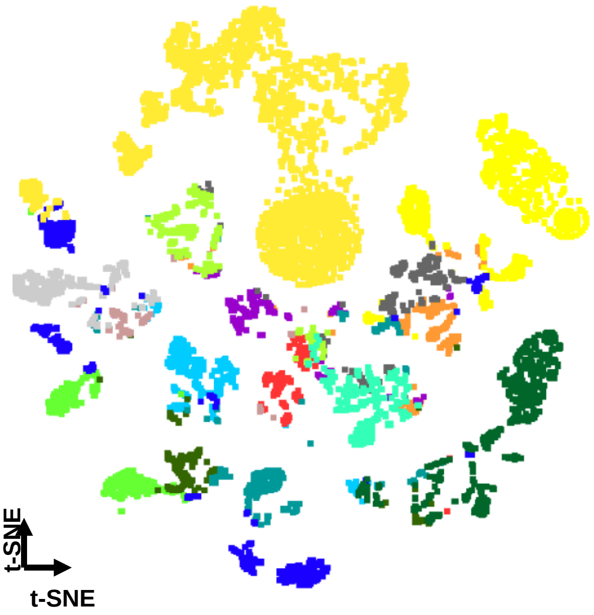

	Subset Name	Count
	F 16	1485
	F 15	769
	F 14	571
	F 13	4666
	F 12	1011
	F 11	761
	F 10	419
	F 9	2184
	F 8	576
	F 7	386
	F 6	148
	F 5	305
	F 4	790
	F 3	436
	F 2	377
	F 1	714
	QC	15,597
